# Measles Without Rash During Acute Febrile Illness Surveillance in Tanzania, 2023–2024

**DOI:** 10.1093/cid/ciaf582

**Published:** 2025-10-16

**Authors:** Saning'o Lukumay, Domitila Augustino, Queen Saidi Naumanga, S Taylor Payne, Godfrey Guga, Joshua Mungi, Mariamu Temu, Kelvin Msoka, Restituta Mosha, Lea Becker, Jie Liu, Esto Mduma, Eric R Houpt

**Affiliations:** Haydom Lutheran Hospital, Haydom Global Health Institute, Haydom, Tanzania; Haydom Lutheran Hospital, Haydom Global Health Institute, Haydom, Tanzania; Division of Infectious Diseases and International Health, University of Virginia, Charlottesville, Virginia, USA; Division of Infectious Diseases and International Health, University of Virginia, Charlottesville, Virginia, USA; Haydom Lutheran Hospital, Haydom Global Health Institute, Haydom, Tanzania; Haydom Lutheran Hospital, Haydom Global Health Institute, Haydom, Tanzania; Haydom Lutheran Hospital, Haydom Global Health Institute, Haydom, Tanzania; Haydom Lutheran Hospital, Haydom Global Health Institute, Haydom, Tanzania; Haydom Lutheran Hospital, Haydom Global Health Institute, Haydom, Tanzania; Division of Infectious Diseases and International Health, University of Virginia, Charlottesville, Virginia, USA; Department of Microbial Surveillance and Biosafety, Qingdao University School of Public Health, Qingdao, China; Haydom Lutheran Hospital, Haydom Global Health Institute, Haydom, Tanzania; Division of Infectious Diseases and International Health, University of Virginia, Charlottesville, Virginia, USA

**Keywords:** measles, rash, fever

## Abstract

**Background:**

The leading causes of fever requiring hospital admission in rural sub-Saharan Africa remain understudied, particularly using modern molecular diagnostics.

**Methods:**

We enrolled 258 patients with acute fever (cases) and 48 controls admitted to 1 hospital in Tanzania for 1 year starting in February 2023. Blood was tested by blood culture, polymerase chain reaction (PCR) for 35 pathogens, and serology for 6 pathogens.

**Results:**

The leading pathogen detected in cases was measles, with 70 of 258 cases detected by PCR, 74 of 238 by immunoglobulin M serology, and 91 allowing either method. Other pathogens were detected in <5% of cases including, in descending order, *Plasmodium*, *Schistosoma*, and *Bartonella*, among others. Measles was the only pathogen clearly associated with cases versus controls. Of 91 measles cases, only 58 (64%) were clinically suspected and only 56 (62%) met the World Health Organization definition of a clinical measles case, largely because 27 (30%) measles cases did not have recorded rash. Outcomes were poorer for measles without rash versus with rash, with a longer hospital stay (mean, 5.5 vs 3.3 days; *P* = .010), less full recovery (48% vs 75%; *P* = .016), and high mortality (7% vs 2%; *P* = .20). Increased laboratory testing would have detected measles 2 months prior to the first clinical suspicion.

**Conclusions:**

Measles was found to be the leading cause of fever during 1 year of acute febrile illness surveillance in rural Tanzania. Measles can present without notable rash in these settings and has poor outcomes. Increasing laboratory test availability and clinician suspicion for measles is a priority.

## Teaser

Measles was the leading diagnosis of acute febrile illness during a year of surveillance in a Tanzanian hospital. Rash was not apparent in 30% of cases, which challenged early detection.

Fever, or acute febrile illness (AFI), is a common reason to present for healthcare, and infections are the cause of >20% of hospital admissions in Africa [1–6]. However, the causes of AFI remain poorly defined, in part because it does not have an accepted case definition. Prior studies have defined fever subjectively or objectively, with different criteria, different durations, with or without particular features or localizing signs [7].

World Health Organization (WHO) guidance for managing febrile illness is also lacking. The assessment of fever in documents such as the Integrated Management of Childhood or Adolescent and Adult Illness [8, 9] emphasizes management of focal infections such as pneumonia and testing or treating malaria [10]. However, if sources are unclear and malaria testing is negative, recommendations are limited. There are myriad potential causes of AFI, most of which are clinically indistinguishable [5, 11–13]. For instance, we examined a series of fever in children from Madagascar, Burkina Faso, and Sudan and found that clinical signs such as cough, abdominal pain, rash, and headache were not sensitive or specific for specific infectious etiologies [13].

Laboratory diagnostics could be of great value; however, tools such as blood culture, serology, and molecular diagnostics are costly and often not available in low-income settings. Many diagnostics are also of low yield; for instance, in large rigorous series from Africa, <4% of fever cases in Africa were blood culture positive for a pathogen [13, 14]. Furthermore, in most studies, diagnostics are only applied to fever patients, not matched controls; therefore, the predictive value of a positive test is not always certain. In some regions of sub-Saharan Africa, about 30% of healthy individuals or patients without fever have detectable malaria [15, 16].

For these reasons, we elected to perform a case-control study over a year of hospital admissions in Tanzania. We allowed a broad case definition of fever, excluding only those with visible wounds or malaria rapid test positive. We hypothesized there would be a wide diversity of pathogens and focused the diagnostic testing to blood, including blood culture, molecular diagnostics, and serologic testing. We utilized a customized TaqMan Array Card (TAC) to perform real-time polymerase chain reaction (PCR) for 35 potential causative pathogens [13, 17, 18]. Serologic testing included testing of acute and convalescent sera for leading potential causes including measles, *Schistosoma*, dengue, *Rickettsia*, and *Brucella*.

## METHODS

### Patient Enrollment

Patients aged >2 years who were admitted to Haydom Lutheran Hospital with an axillary temperature of ≥37.5°C and whose illness symptoms started <7 days prior to admission were eligible for enrollment. The hospital has 420 beds and serves a large rural catchment area. There is very low malaria transmission in the region because of high altitude (1753 meterselevation). Our team enrolled patients Monday through Saturday during working hours. We excluded those with positive rapid malaria tests (n = 9) and those with wound/skin infections (n = 2). For every fifth case, we enrolled a control without fever who was sex and age matched to within 10 years of age and admitted to the same ward with noninfectious diagnoses. Blood was obtained at admission, during hospital day 2–3 when not available upon admission, and at 1 month after enrollment. Admission blood underwent blood culture and PCR. Admission and discharge diagnoses were categorized into the primary diagnosis (Table 1). Chart review and laboratory values were collected observationally from the medical record for all days of hospital admission. Case report forms recorded symptoms in all participants upon admission and discharge. In measles cases, we subsequently reviewed every word of the medical record to assess if rash was ever noted. Each case and control patient underwent follow-up at 1 month after enrollment for questionnaire and venipuncture. The study was reviewed and approved by the Tanzania National Institute for Medical Research and the University of Virginia Institutional Review Board (HSR220151).

**Table 1.** Characteristics of the Study Population

Characteristic	Value
Demographics	
Total cases, No.	258
Female sex	124 (48)
Age, y, median (range)	9 (2–92)
Adult (≥18 y)	90 (35)
Pediatric (<18 y)	168 (65)
Socioeconomic status (WAMI score, range 0–1) [19]	0.35 ± 0.19
Antibiotics prior to admission^a^	65 (28)
Days inpatient	4.8 ± 5.7
Primary clinical diagnosis on discharge^b^	
Measles	62 (24)
Pneumonia/LRTI	37 (14)
Gastrointestinal/abdominal/diarrhea	29 (11)
Tuberculosis	23 (9)
URTI	20 (8)
Sepsis	13 (5)
Meningitis	12 (5)
Other	12 (5)
UTI	11 (4)
CNS/psychiatric	7 (3)
Hematology/oncology	7 (3)
COPD/asthma	6 (2)
HIV-1	6 (2)
Malnutrition	6 (2)
Cardiac	3 (1)
Kidney disease	3 (1)
Antibiotics administered upon admission	236 (91)
Laboratory values	
Temperature on admission, °C	38.4 ± 0.7
White blood cell count, cells/μL	9.9 ± 7.5
Hemoglobin, g/L	11.7 ± 2.5
Outcome at 1 m follow-up	
Full recovery (self-reported)	153 (59)
Partial recovery (self-reported)	73 (28)
No recovery (self-reported)	1 (0.4)
Died	14 (5)
Lost to follow-up	17 (7)

Data are presented as No. (%) or mean ± SD unless otherwise indicated.

Abbreviations: CNS, central nervous system; COPD, chronic obstructive pulmonary disease; HIV-1, human immunodeficiency virus type 1; LRTI, lower respiratory tract infection; URTI, upper respiratory tract infection; UTI, urinary tract infection; WAMI, Water, Assets, Education, Income index.

^a^23 cases did not know if antibiotics were taken prior.

^b^Primary diagnosis was based on discharge diagnoses and was available for n = 257. “Other” included chickenpox, diabetes, hydrocephalus, intoxication, deep vein thrombosis, febrile seizures, foreign body, osteoarthritis, and osteomyelitis.

### Laboratory Methods and PCR

Whole blood was tested using a customized TAC, a 384-well real-time PCR system [20]. Blood for PCR was batch tested at the end of the study. If available blood remained from the 1 month follow-up, this was tested by TAC as well. Primer and probe oligonucleotide sequences were designed to detect 14 bacteria, 13 viruses, 2 fungi, and 6 parasites (Supplementary Figure 1). All assays have been published previously [17, 20–22]. In brief, 2 mL of ethylenediaminetetraacetic acid whole blood was extracted using the High Viral Nucleic Acid Large Volume kit (Roche, Basel, Switzerland) and eluted in 150 μL. Then, 75 μL of the nucleic acid extract was mixed with 25 μL of TaqMan Fast Virus One Step RT-PCR mastermix (Thermo Fisher, Carlsbad, CA, USA) and loaded into the TAC, then subjected to quantitative reverse-transcription PCR. External controls, bacteriophage MS2 and phocine herpesvirus, were spiked into each sample to confirm extraction and amplification fidelity and were required to be positive with a quantification cycle (Cq) <35. One negative control was included in each batch of extractions and was required to be negative with a Cq ≥40 to rule out laboratory contamination. For detections by PCR with a Cq of >35, we required confirmation by repeat PCR in order to be positive.

### Serology

Admission and 1-month follow-up serum samples were batch tested by enzyme-linked immunosorbent assay (ELISA) for *Brucella* immunoglobulin G (IgG) (EUROIMMUN, Lübeck, Germany, E240131RB), *Schistosoma* IgG (DRG international, EIA-3512), dengue IgG (DENV Detect, InBios International, Seattle, WA, USA), and spotted fever group *Rickettsia* IgG and *Rickettsia typhi* IgG (Fuller Laboratories, Fullerton, CA, USA). For measles, admission serum was tested using Anti-Measles Viruses NP ELISA (immunoglobulin M [IgM]) kit (EUROIMMUN). All serologic tests were performed and interpreted according to the manufacturer's instructions. We defined laboratory-confirmed measles as IgM seropositivity and/or detection by PCR, consistent with WHO guidance [23]. For IgG serologies, we defined positive as seroconversion, whereby acute sera were negative/equivocal and convalescent sera were positive for IgG. Convalescent serum negative for IgG was considered negative. When acute serum was positive for IgG, we considered that previously positive and unevaluable. Other scenarios such as if convalescent IgG results were borderline or equivocal per manufacturer's instructions were considered nondiagnostic.

### Statistical Analysis

Means were compared using *t* test. Proportions and 95% confidence intervals (CIs) were calculated using the Wald method. Rates of pathogens detected in cases versus controls were analyzed by Fisher exact test. *P* values were 2 tailed with <.05 considered significant. Statistics were performed using GraphPad software (https://www.graphpad.com/quickcalcs/). Fleiss’ kappa for agreement among PCR, IgM, and clinical suspicion was performed using rBiostatistics (https://rbiostatistics.com/node/67).

## RESULTS

We enrolled 258 cases of AFI and 48 controls over the 12-month period from February 2023 to February 2024. The study population is summarized in Table 1. Enrollment occurred 6 days a week and was 17–27 cases per month. Forty-eight percent of cases were female, 35% were adults, and 65% were <18 years of age. The socioeconomic status of the population was low (Water, Assets, Education, Income index [WAMI] score 0.35 [19]). The 3 leading clinician-assessed discharge diagnoses were measles (24%), pneumonia/lower respiratory tract infection (14%), and gastrointestinal/abdominal/diarrhea (11%). At 1-month follow-up, 5% had died, 28% reported partial recovery, and 59% reported full recovery.

### PCR and Serological Results

All AFI cases had blood collected upon enrollment for PCR using a customized TAC with assays to detect 35 pathogens. The leading pathogen detected in cases was measles (70 [27%]), followed by *Plasmodium*, *Schistosoma*, and *Bartonella* (each 3%–4%), followed by *Rickettsia*, *Coxiella*, *Brucella*, *Streptococcus pneumoniae*, dengue, hepatitis E, and *Borrelia* (≤2% each) (Table 2). In total, 39% of cases had 1 or more pathogens detected by PCR compared with only 12% of controls (*P* < .0009). Measles PCR positivity was highly associated with AFI case status (27% vs 2% in controls; *P* < .0001).

**Table 2.** Results of Blood Polymerase Chain Reaction, Blood Culture, and Serology Upon Enrollment

Test and Pathogen	Cases (n = 258)	Controls (n = 48)
PCR: No. with pathogen(s) detected^a^	100 (39)^b^	7 (12)
Measles	70 (27)^b^	1 (2)
*Plasmodium* spp.	10 (4)	3 (6)
*Schistosoma* spp,	9 (3)	0 (0)
*Bartonella* spp.	8 (3)	3 (6)
*Rickettsia* spp,	5 (2)	1 (2)
*Coxiella burnetii*	4 (2)	0 (0)
*Brucella* spp.	3 (1)	0 (0)
*Streptococcus pneumoniae*	3 (1)	0 (0)
Dengue	1 (0.4)	0 (0)
Hepatitis E	1 (0.4)	0 (0)
*Borrelia* spp.	1 (0.4)	0 (0)
PCR: No. with no pathogen detected	158 (61)	41 (88)
Blood culture positive	9 (3)	1 (2)
*Staphylococcus aureus*	6 (2)	1 (2)
*Escherichia coli*	3 (1)	0 (0)
Serology		
Measles IgM^c^	74 (31)^b^	7 (15)
*Brucella* IgG seroconversion^d^	2 (1)	0 (0)
*Rickettsia* spotted fever group IgG seroconversion^e^	7 (5)	1 (4)
*Rickettsia* Typhus group IgG seroconversion^f^	16 (11)	5 (19)
*Schistosoma mansoni* IgG seroconversion^g^	14 (9)	3 (10)
Dengue IgG seroconversion^h^	5 (3)	1 (4)
Any pathogen using methods above	122 (47)	16 (33)
No pathogen using methods above	136 (53)	32 (66)

Data are presented as No. (%).

Abbreviations: IgG, immunoglobulin G; IgM, immunoglobulin M; PCR, polymerase chain reaction.

^a^86 (33%) had 1 pathogen detected by PCR; 14 (5%) had >2 pathogens detected, led by measles + *Plasmodium* (n = 6).

^b^ *P* < .05, rate of detection of pathogens in cases versus controls.

^c^No. excludes 20 cases and 1 control without enrollment sera and 3 cases and 1 control with borderline results.

^d^116 had preexisting IgG, nondiagnostic enzyme-linked immunosorbent assay (ELISA) results, or unavailable sera.

^e^187 had preexisting IgG, nondiagnostic ELISA results, or unavailable sera.

^f^194 had preexisting IgG, nondiagnostic ELISA results, or unavailable sera.

^g^246 had preexisting IgG, nondiagnostic ELISA results, or unavailable sera.

^h^124 had preexisting IgG, nondiagnostic ELISA results, or unavailable sera.

Blood culture was positive in 9 cases: 6 with *Staphylococcus aureus* and 3 *Escherichia coli*. Acute sera were available from most cases (238/258 [93%]) and 74 of 238 (31%) were positive for measles IgM, compared with 7 of 46 (15%) controls (*P* = .03). Available acute and convalescent sera were tested for *Brucella, Rickettsia* spotted fever group, *Rickettsia* Typhus group, *Schistosoma mansoni*, and dengue IgG seroconversion. Rates of IgG seroconversion for each of these pathogens was similar between cases and controls (*P* = .56–1.0). IgG seroconversions and blood culture results were examined in measles versus nonmeasles cases and were generally similar in both groups (Supplementary Table 1). Agreement between measles PCR and IgM was 85% in the 235 cases that had results for both assays. For other pathogens, the correlations between serology and PCR were poor (all IgG seroconversions were PCR negative for the relevant pathogen except for 1 *Rickettsia*; Supplementary Table 2). In total, accepting any of the above test methods, 122 cases (47%) had any pathogen found compared with 16 controls (33%; *P* = .08). Of the 14 deaths, 3 had measles, 1 had *S. aureus* in blood culture, and the remainder had no pathogen detected. Cases who died were older than those who survived (mean, 40 ± 33 years [95% CI, 21–59] vs 18 ± 19 years [95% CI, 15–20]; *P* < .0001).

### Clinical Presentations of Measles

**Figure 1.**
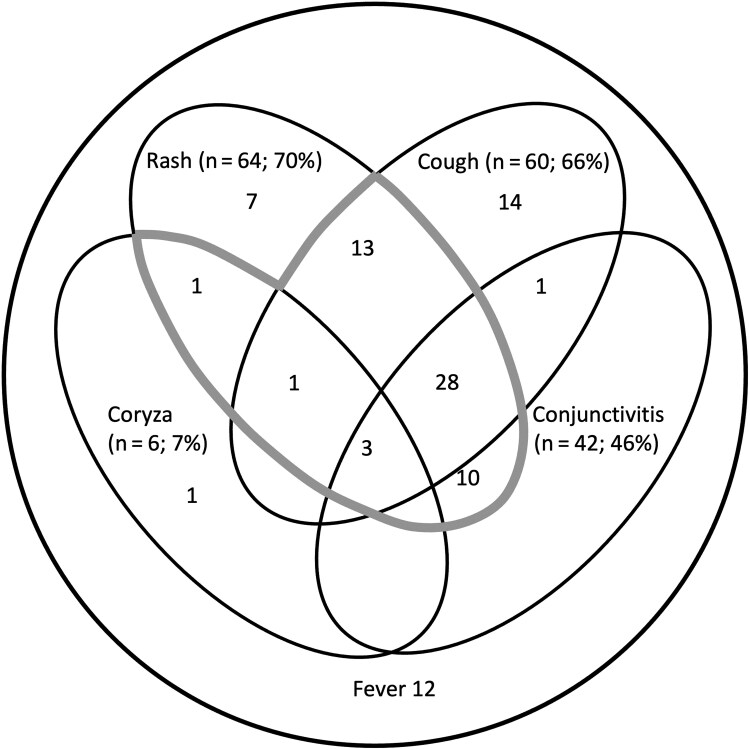
Symptoms among measles cases during hospital admission. Symptoms of fever, rash, cough, coryza, and conjunctivitis and combinations thereof are shown for all 91 laboratory-confirmed cases of measles (black numbers = n). The World Health Organization definition of a clinical case of measles is fever plus rash and 1 of cough, coryza, and conjunctivitis (outlined in gray), n = 56.

Measles was the leading diagnosis in this AFI study, with 91 of 258 (35%) cases with laboratory-confirmed measles infection, defined as IgM or PCR positive per WHO [23]. We examined the clinical presentations of these cases (Table 3). There were 67 clinician-suspected measles cases (26%), either as the admission or discharge diagnosis. The overlap between clinical suspicion, PCR, and IgM results was substantial, with a kappa agreement of 0.63 (Supplementary Figure 2). The WHO defines a clinical case of measles as fever plus rash plus at least 1 of cough, coryza, and conjunctivitis, and 60 individuals (23%) met this definition [23]. Therefore, clinician suspicion was 64% sensitive and WHO clinical criteria were 62% sensitive for laboratory-confirmed measles. The low sensitivity of clinical case criteria was because 30% (27/91) of laboratory-confirmed cases did not have rash recorded by the study teams nor documented in the medical record; that is, only 70% of laboratory-confirmed measles cases had rash. The combinations of all pertinent measles symptoms are shown in Figure 1. The most common scenario was a patient with fever, rash, conjunctivitis, and cough (28/91 [31%]), followed by fever and cough only (14/91 [15%]). There were 12 cases (13%) with fever and no other signs. Coryza was noted in only 7% of cases. Of the 38 laboratory-confirmed measles cases that were not clinically suspected, the primary clinical diagnoses were pneumonia/lower respiratory tract infection (13%), followed by tuberculosis (5%), urinary tract infection (5%), gastrointestinal/abdominal/diarrhea (3%), meningitis (3%), and several other diagnoses (Supplementary Table 3). Among clinical diagnoses besides measles, pathogen detection was low, with only 67 of 196 (34%) nonmeasles clinical diagnoses having a pathogen detected across a range of clinical diagnoses (Supplementary Table 4).

**Table 3.** Clinical Features Among Confirmed Measles Versus Nonmeasles Cases

Feature	Laboratory-Confirmed Measles, No.	Not Measles, No.	Sensitivity	Specificity
Clinical suspicion of measles	58	9	64%	95%
No clinical suspicion	33	158		
WHO clinical case criteria: Fever plus rash plus 1 of cough, coryza, or conjunctivitis	56	4	62%	98%
No WHO clinical case criteria	35	163		
Rash^a^	64	7	70%	96%
No rash	27	160		

Abbreviation: WHO, World Health Organization.

^a^Rash could have been reported at any time from admission to discharge.

We compared measles cases with rash to those without rash (Table 4). Measles patients without rash were typically older (mean age, 22 vs 12 years; *P* = .0008) and had a longer inpatient stay (mean, 5.5 vs 3.3 days; *P* = .010), a high mortality (7% vs 2%; *P* = not significant), and less full recovery (46% vs 75%; *P* = .016) than measles cases with rash. Measles cases with rash were more likely to be PCR positive (88%), IgM positive (90%), and positive by both methods (76%). By contrast, only 22% of measles cases without rash were positive by both methods (*P* = .0001).

**Table 4.** Clinical Features of Measles With Versus Without Rash

Feature	Measles With Rash	Measles Without Rash	*P* Value
Total cases, No.	64	27	
Female sex	28 (44)	15 (56)	.36
Age, y, mean (95% CI)	12 (10–14)	22 (15–29)	.0008
Temperature	38.3 ± 0.7	38.5 ± 0.8	.10
Days inpatient, mean (95% CI)	3.3 (2.9–3.7)	5.5 (3.0–8.0)	.010
Mortality at 1 m	1 (2)	2 (7)	.20
Full recovery at 1 m	48 (75)	13 (48)	.016
Measles PCR positive	56/64 (88)	14/27 (52)	.0007
Cq value	28.7 ± 3.2	28.8 ± 3.6	.95
Measles IgM positive^a^	55/61 (90)	19/27 (70)	.027
Measles PCR and IgM positive^a^	47/62 (76)	6/27 (22)	.0001

Data are presented as No. (%) unless otherwise indicated.

Abbreviations: CI, confidence interval; Cq, quantification cycle; IgG, immunoglobulin G; IgM, immunoglobulin M; PCR, polymerase chain reaction.

^a^3 sera did not have IgM results (2 did not have sera and 1 was borderline).

**Figure 2.**
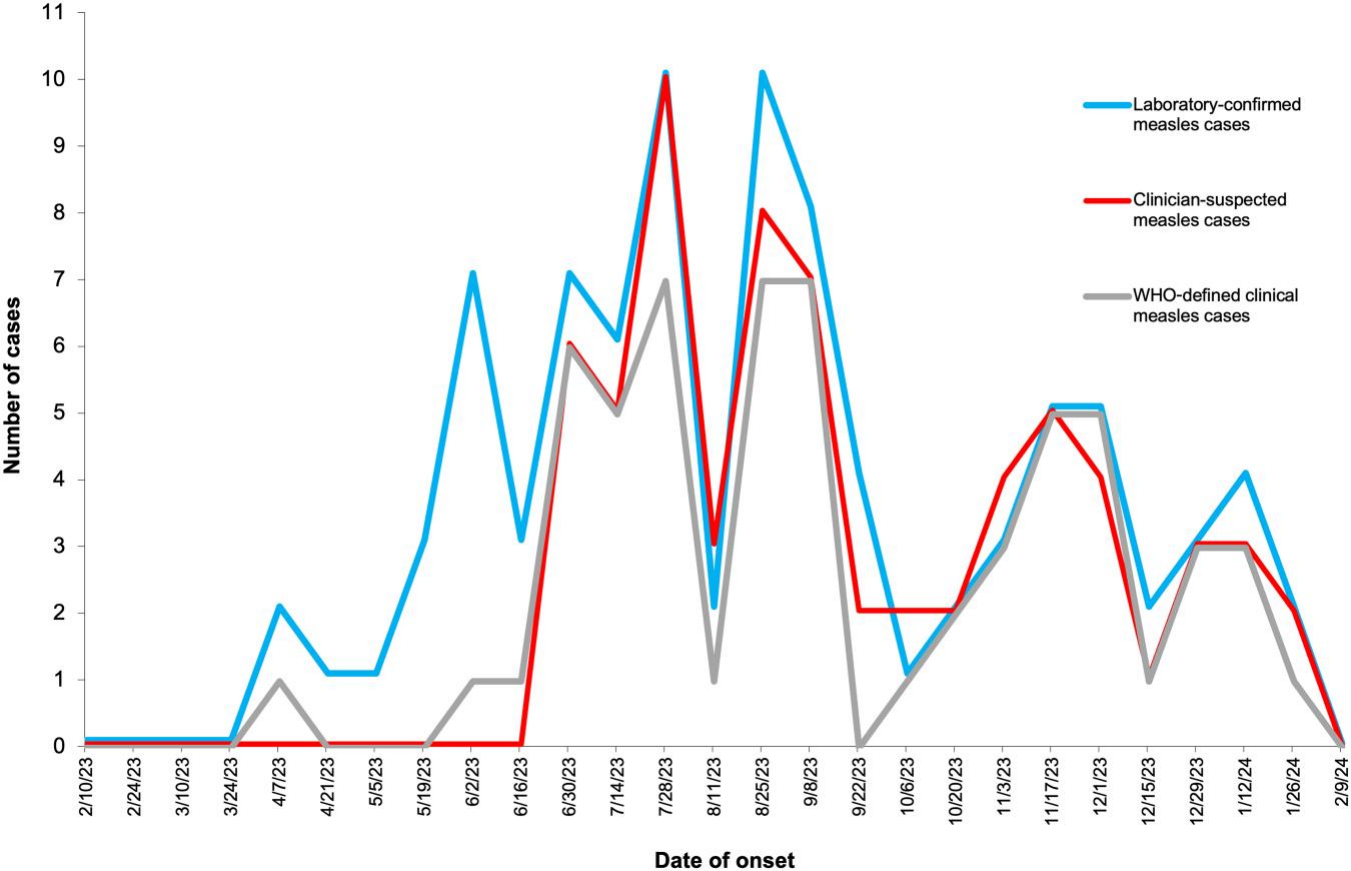
Epidemic curve of measles cases. The dates of onset of laboratory-confirmed, clinician-suspected, and World Health Organization (WHO)–defined clinical measles cases are shown.

The epidemic curve showed that the first clinical suspicion of measles and the first WHO-defined measles case was delayed compared with the first case identified by laboratory testing (Figure 2). Laboratory testing was first positive in April 2023 and was positive in 17 more cases before the first measles clinical suspicion in June 2023. Of these first 17 clinician-unsuspected laboratory-confirmed measles cases, 76% (13/17) had no rash. The 4 cases where rash was noticed were diagnosed as marasmus rash or dermatitis. Of the 17 early laboratory detections, there was no clear difference in detection methodology: 10 were detected by PCR, 14 by serology, and 7 by both methods.

## DISCUSSION

The key finding from this year of AFI surveillance in a rural Tanzanian setting was the high rate of measles. Certainly measles is known to occasionally occur in the region and the country has a case-based surveillance system [24]; however, this rate was unexpectedly high, with 35% of a year's enrolled hospital admissions with fever having laboratory-confirmed measles.

The high prevalence of measles was presumably happenstance in that our study coincided with a measles outbreak in the area. The hospital's medical record registry confirms that 2023 had particularly high measles admissions compared with 2022 or 2024. At the time of our study, clinically suspected measles cases were reported to the local authorities and local vaccine campaigns were implemented, which may have helped reduce the outbreak [24]. The coincidence of our AFI surveillance with a measles outbreak allowed for a rigorous assessment of clinical presentations and diagnostic performance in a real-world setting. The clinical presentations of measles are important to reassess given the reemergence of measles.

We found that rash was noted in only 70% of measles cases. Rash is considered a critical characteristic for measles, and case definitions require fever plus rash. Most literature reviews describe the typical time course of fever and rash but do not mention measles without rash [25]. Rash is typically described as appearing a few days after onset of fever. Since we enrolled participants within 7 days of fever initiation, and since cases were on average admitted for 4.8 days, there was ample time for the typical time course of rash to present during our data collection. Certainly, it is possible that a rash was minimal, missed because of the darker-skinned population, not reported by the patient, or simply missed by the clinicians or our study teams; however, we suspect most of these individuals truly did not manifest rash. Measles without rash has been reported before, including in severe and fatal cases [26, 27]. In our study the clinical outcomes were serious for measles and for measles without rash in particular, with a longer length of stay, less recovery, and high mortality. We suggest that other hospitals experiencing measles outbreaks assess whether cases may also be presenting without rash.

It is possible that many of our cases had partial immunity from prior vaccination or earlier disease, which can lead to atypical presentations. Many of the cases without rash were older than is typical for measles in Tanzania, with an average age of 22 years [24]. Unfortunately, we could not obtain data on immunization history from our participants, as children usually present to hospital without their vaccine cards and the ability to document individual historical vaccination history is limited. Understanding whether cases occurred in fully, partially, or unvaccinated individuals would be valuable to guide vaccine strategies.

Another feature of our study was that laboratory detection preceded clinical detection. Laboratory testing identified measles in April 2023 while the first clinical diagnosis occurred in late June. A system of routine laboratory testing could have identified the outbreak earlier and could have guided hospital infection control efforts; therefore, increasing the availability of diagnostics for measles is an important goal. We found a reasonable 85% agreement between IgM testing and PCR testing of blood. Interestingly, we noted a clearer association between AFI and measles PCR positivity than IgM testing in cases versus controls, suggesting that PCR may be more specific and some IgM-positive results could be false. Alternatively, it is well known that PCR and serology can be discordant: Hyperacute cases can be PCR positive and not yet IgM positive, and likewise measles can be IgM positive but no longer viremic and PCR negative. The observation that many measles cases without rash were positive with only 1 method and not both could reflect these cases presenting very early or late in their course or indicate some false positives.

We performed blood culture and IgG serology for several other pathogens. Blood culture was particularly low yield (3% positive). Unsurprisingly, 91% of cases presented already on antibiotics. Performing blood culture is costly and resource intensive, and the low rate of results dampens enthusiasm for uptake in our setting. Serological results were also mixed. Many patients had nondiagnostic results because of having previously positive IgG at baseline or borderline results. Correlations between serology and PCR were poor. Many seroconversions were seen in controls, notably Typhus group *Rickettsia* and *Schistosoma*. Finally, many seroconversions were seen in measles cases, where laboratory results and clinician assessment provided a more convincing diagnosis.

Acknowledging these uncertainties in causality, there did appear to be a broad diversity of pathogens beyond measles, including malaria, *Schistosoma*, *Bartonella*, *Rickettsia*, *Coxiella*, and *Brucella*. We believe that better diagnostic methods beyond whole blood PCR and serology are needed, along with improved study designs and clinical algorithms to guide the AFI laboratory workup. Even if one generously accepts that many of the pathogen detections in this study were clinically relevant, the undiagnosed fraction with no pathogens detected remained high at 53%.

This study had several limitations. We could not ascertain measles vaccine status, which limits our understanding of the outbreak. We did not test other specimens such as respiratory specimens, stool, urine, or cerebrospinal fluid, and we could have expanded the test portfolio to better target the clinical diagnoses. We did not have available sera from all cases, and we only performed ELISA methods. This limited serologic assessments with PCR. Detection of pathogens directly in blood by PCR is intrinsically challenged by the low number of pathogens present, particularly for bacteria [20]. It is unclear whether these findings of measles without rash were specific to this particular geography and epidemic context or will be found to be more generalizable.

In conclusion, clinicians should be alert to the possibility of measles presenting without rash or prominent rash. We found laboratory testing to be the most reliable method for detecting measles. Availability of laboratory testing for measles, including development of measles rapid diagnostic tests, should be scaled up. This would allow greater detection of measles, infection control measures to mitigate spread, and timely institution of vaccination initiatives [23].

## Supplementary Material

ciaf582_Supplementary_Data

## Supplementary Data

Supplementary materials are available at *Clinical Infectious Diseases* online. Consisting of data provided by the authors to benefit the reader, the posted materials are not copyedited and are the sole responsibility of the authors, so questions or comments should be addressed to the corresponding author.
