# Supplementary material for: Measles Without Rash During Acute Febrile Illness Surveillance in Tanzania, 2023–2024
Source: Clin Infect Dis. 2025 Oct 16;82(6):e1208–15. doi: 10.1093/cid/ciaf582 (PMC13341257; doi:10.1093/cid/ciaf582)
Supplement: ciaf582_Supplementary_Data [file ciaf582_supplementary_data.docx]

**Supplemental Figure 1.** Customized TAC card for this acute febrile illness study


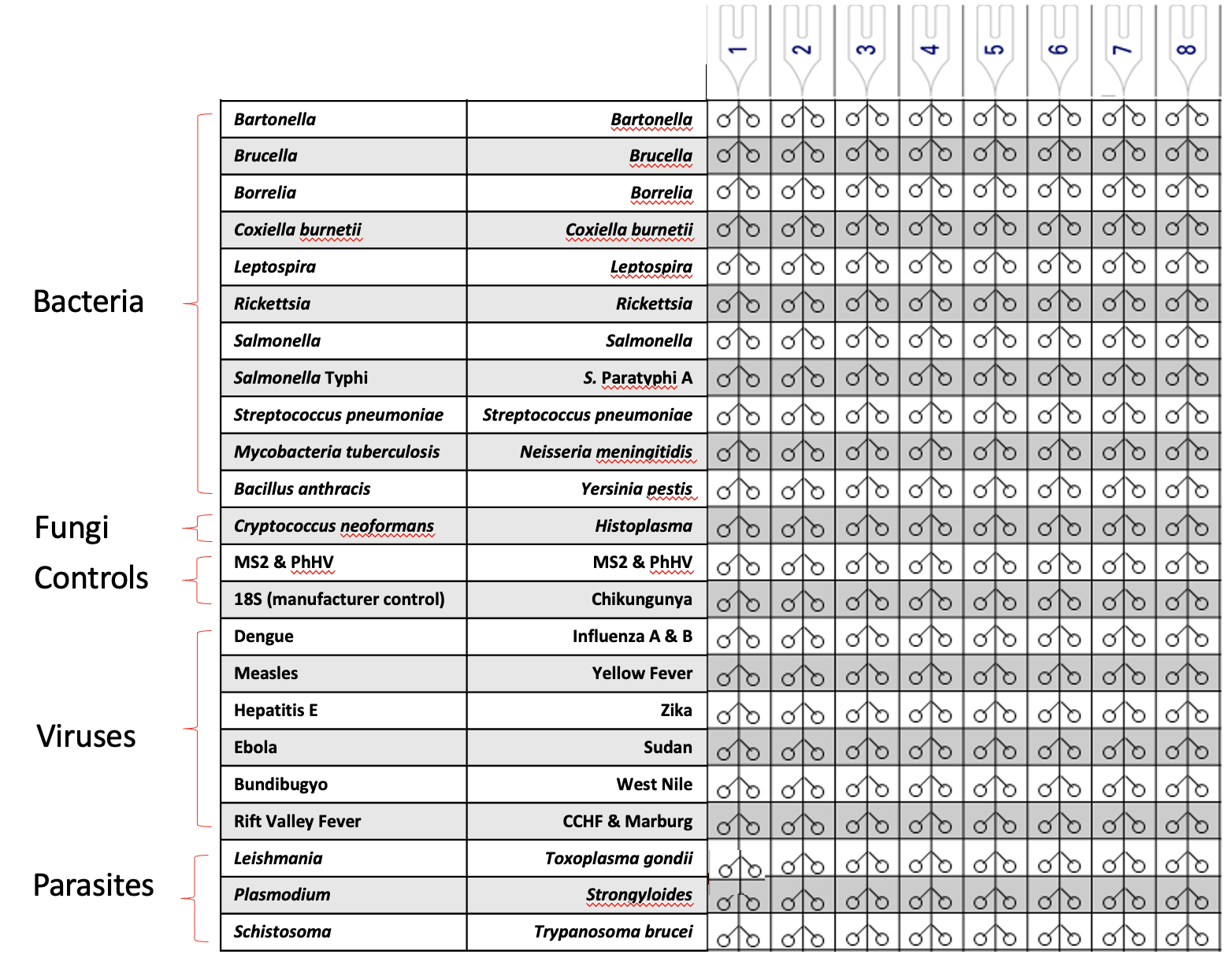


**Supplemental Figure 2.** Agreement between PCR, IgM, and clinical suspicion for measles among acute febrile illness cases. Excludes 23 cases without sera results.


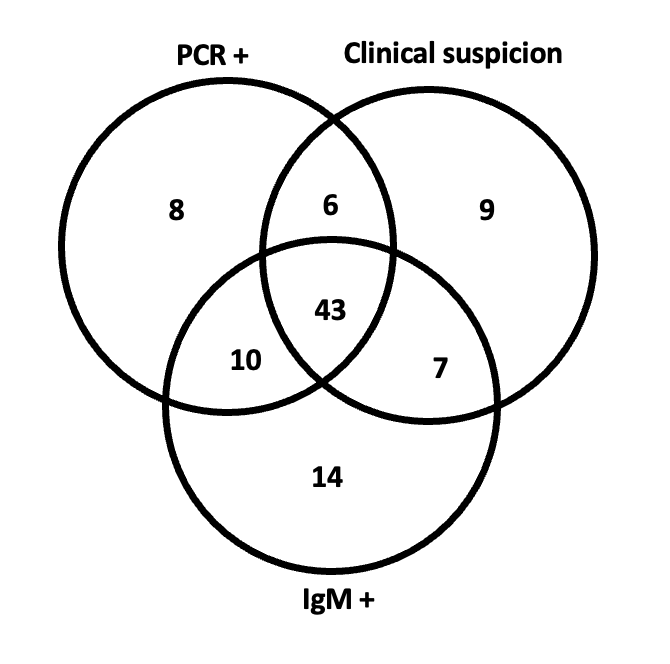


**Supplemental Table 1.** Blood culture and serologic detections among measles cases

|  | In cases with lab-confirmed measles^a^ | In cases without measles (laboratory negative) ^b^ |
| --- | --- | --- |
| Blood culture positive | 1 (1%) | 1 (2%) |
| *S. aureus* | 1 (1%) | 5 (3%) |
| *E. coli* | 0 (0%) | 3 (2%) |
| *Brucella* IgG seroconversion^a^ | 2 (3%) | 0 (0%) |
| *Rickettsia* Spotted Fever Group IgG seroconversion^a^ | 5 (7%) | 2 (2%) |
| *Rickettsia* Typhus Group IgG seroconversion^a^ | 7 (10%) | 9 (10%) |
| *Schistosoma* mansoni IgG seroconversion^a^ | 8 (11%) | 6 (7%) |
| Dengue IgG seroconversion^a^ | 1 (1%) | 4 (4%) |

^a^73/91 (80%) measles cases had available IgG results

^b^91/167 (54%) non-measles cases had available IgG results

**Supplemental Table 2.** Correlation between serological results and PCR for non-measles pathogens

*Brucella* spp.^a^

|  | PCR+ | PCR- |
| --- | --- | --- |
| IgG seroconversion | 0 | 2 |
| IgG negative | 0 | 188 |

^a^ Could not evaluate 3 individuals with pre-existing IgG, 1 with nondiagnostic ELISA results, and 112 with unavailable sera

*Rickettsia* Spotted Fever Group^b^

|  | PCR+ | PCR- |
| --- | --- | --- |
| IgG seroconversion | 0 | 8 |
| IgG negative | 2 | 109 |

^b^ Could not evaluate 49 individuals with pre-existing IgG, 26 with nondiagnostic ELISA results, and 112 with unavailable sera

*Rickettsia* Typhus Group ^c^

|  | PCR+ | PCR- |
| --- | --- | --- |
| IgG seroconversion | 1 | 20 |
| IgG negative | 1 | 90 |

^c^ Could not evaluate 56 individuals with pre-existing IgG, 26 with nondiagnostic ELISA results, and 112 with unavailable sera

*Schistosoma mansoni*^d^

|  | PCR+ | PCR- |
| --- | --- | --- |
| IgG seroconversion | 0 | 17 |
| IgG negative | 2 | 41 |

^d^ Could not evaluate 133 individuals with pre-existing IgG, 6 with nondiagnostic ELISA results, and 107 with unavailable sera

Dengue

|  | PCR+ | PCR- |
| --- | --- | --- |
| IgG seroconversion | 0 | 6 |
| IgG negative | 1 | 175 |

^e^ Could not evaluate 3 individuals with pre-existing IgG, 8 with nondiagnostic ELISA results, and 113 with unavailable sera

**Supplemental Table 3.** Primary clinical diagnoses upon discharge among 91 laboratory-confirmed measles cases

| **Primary Clinical Diagnosis** |  |
| --- | --- |
| Measles | 53 (58%) |
| Pneumonia/LRTI | 12 (13%) |
| Tuberculosis | 5 (5%) |
| UTI | 5 (5%) |
| Gastrointestinal/Abdominal/Diarrhea | 3 (3%) |
| Meningitis | 3 (3%) |
| HIV | 2 (2%) |
| Cardiac | 2 (2%) |
| Sepsis | 1 (1%) |
| Other | 1 (1%) |
| Hematology/Oncology | 1 (1%) |
| COPD/Asthma | 1 (1%) |
| Malnutrition | 1 (1%) |
| Kidney disease | 1 (1%) |

**Supplemental Table 4.** Rates of pathogen detection by primary clinical diagnosis. Pathogens could be positive by PCR, blood culture, IgM, or IgG seroconversion according to the methods

| **Clinical Diagnosis** | **N with 1 or more pathogen detected (%)** |
| --- | --- |
| Measles | 55/62 (89%) |
| Non-Measles | 67/196 (34%) |
| Pneumonia/LRTI | 18/37 (49%) |
| Gastrointestinal/Abdominal/Diarrhea | 10/29 (34%) |
| Tuberculosis | 8/23 (35%) |
| URTI | 2/20 (10%) |
| Sepsis | 4/13 (30%) |
| Meningitis | 4/12 (33%) |
| Other | 1/12 (8%) |
| UTI | 5/11 (45%) |
| CNS/Psychiatric | 3/7 (42%) |
| Hematology/Oncology | 1/7 (14%) |
| COPD/Asthma | 2/6 (33%) |
| HIV | 2/6 (33%) |
| Malnutrition | 4/6 (66%) |
| Cardiac | 2/3 (66%) |
| Kidney disease | 1/3 (33%) |
| Total | 122 (47%) |
